# Zoonotic infection of Brazilian primate workers with New World simian foamy virus

**DOI:** 10.1371/journal.pone.0184502

**Published:** 2017-09-20

**Authors:** Cláudia P. Muniz, Liliane T. F. Cavalcante, Hongwei Jia, HaoQiang Zheng, Shaohua Tang, Anderson M. Augusto, Alcides Pissinatti, Luiz P. Fedullo, André F. Santos, Marcelo A. Soares, William M. Switzer

**Affiliations:** 1 Departamento de Genética, Universidade Federal do Rio de Janeiro, Rio de Janeiro, Brazil; 2 Laboratory Branch, Division of HIV/AIDS Prevention, Centers for Disease Control and Prevention, Atlanta, Georgia, United States of America; 3 Fundação Jardim Zoológico da Cidade do Rio de Janeiro, Rio de Janeiro, Brazil; 4 Centro de Primatologia do Rio de Janeiro, Rio de Janeiro, Brazil; 5 Programa de Oncovirologia, Instituto Nacional de Câncer, Rio de Janeiro, Brazil; University of Pittsburgh Centre for Vaccine Research, UNITED STATES

## Abstract

Simian foamy viruses (SFVs) are retroviruses present in nearly all nonhuman primates (NHPs), including Old World primates (OWP) and New World primates (NWP). While all confirmed human infections with SFV are from zoonotic transmissions originating from OWP, little is known about the zoonotic transmission potential of NWP SFV. We conducted a longitudinal, prospective study of 56 workers occupationally exposed to NWP in Brazil. Plasma from these workers was tested using Western blot (WB) assays containing NWP SFV antigens. Genomic DNA from blood and buccal swabs was analyzed for the presence of proviral SFV sequences by three nested PCR tests and a new quantitative PCR assay. Exposure histories were obtained and analyzed for associations with possible SFV infection. Ten persons (18%) tested seropositive and two persons were seroindeterminate (3.6%) for NWP SFV. Six persons had seroreactivity over 2–3 years suggestive of persistent infection. All SFV NWP WB-positive workers reported at least one incident involving NWP, including six reporting NWP bites. NWP SFV viral DNA was not detected in the blood or buccal swabs from all 12 NWP SFV seroreactive workers. We also found evidence of SFV seroreversion in three workers suggestive of possible clearance of infection. Our findings suggest that NWP SFV can be transmitted to occupationally-exposed humans and can elicit specific humoral immune responses but infection remains well-controlled resulting in latent infection and may occasionally clear.

## Introduction

Emerging infectious diseases in humans are an important public health concern with most having a zoonotic origin and over 70% originating from wildlife [1]. Recent examples of emerging infectious diseases with high public health significance include the Ebola virus outbreak in West Africa and the Zika virus outbreak in Central and South America. Among retroviruses, simian immunodeficiency viruses (SIVs) and simian T-cell lymphotropic viruses (STLVs) are prevalent in nonhuman primates (NHPs), and crossed into and spread amongst humans to become the human immunodeficiency viruses (HIV) and human T-lymphotropic viruses (HTLV), respectively (2). Similarly, simian foamy virus (SFV) is another complex retrovirus that is highly prevalent in NHPs, including Old Word primates (OWP) and New World primates (NWP) that can zoonotically infect humans [2]. All confirmed SFV infections identified so far in humans have resulted from zoonotic transmission of SFV infecting OWPs [3].

SFV DNA integrates as a provirus in the genomic DNA of many tissues and organs from infected animals, including peripheral blood mononuclear cells (PBMCs), lung, kidney and liver [4, 5]. However, SFV replication has been reported to be limited to superficial epithelial cells of the oral mucosa [6–9], supporting horizontal transmission through bites as the major route of SFV infection. Humans infected with SFV have been reported in occupationally and naturally exposed individuals with direct contact with NHPs and/or their body fluids, including veterinarians, zoo keepers, hunters and butchers [10–15]. SFV seroprevalence in exposed humans can reach 37%, depending on the studied population and the involved risk activity, with persons reporting severe bites having greater risk of infection [9, 13, 16–19]. SFV DNA and antibodies can be detected in both blood and saliva of infected humans, but there are no reports of viral RNA expression at those sites [20–23]. So far, evidence of SFV transmission between humans has not been described [17].

SFV is known for its long persistence and non-pathogenic effects in infected mammalian hosts, including zoonotically infected humans, despite the cytopathic effects and cellular death observed in infected cell cultures (21, 22). Nonetheless, most studies have focused on infection of healthy workers and hunters and it is not known what effects SFV may have in unhealthy persons or immunocompromised hosts. Co-infection with SFV and HIV-1 has been characterized in a commercial sex worker from the Democratic Republic of Congo, a blood donor from Cameroon, and two persons from Ivory Coast [24, 25] raising questions about possible SFV pathogenicity in human hosts immunocompromised by HIV [24, 25]. Interestingly, rhesus macaques co-infected with simian immunodeficiency virus (SIV) and SFV show increases in SIV plasma viral loads, faster CD4^+^ T-cell decline and accelerated progression to simian AIDS compared with SIV mono-infected macaques [26]. Combined, these results suggest that further studies are needed to define the pathogenic potential of SFV.

SFVs have been identified in many species of all three major NWP (Platyrrhini) families (Cebidae, Atelidae, Pitheciidae) in both captive and wild-caught monkeys with high prevalences in some species (32–35). In addition, NWPs are common members of zoological collections, are frequently used in research studies [27], and are hunted in the wild by Amerindians and other groups and kept as pets or for consumption [28]. NWP SFV can also infect a variety of human cells *in vitro* (34). Combined, these findings suggest that humans in contact with NWPs may be at risk for infection with SFV as has been shown for OWP exposures. A single cross-sectional study by Stenbak *et al*. detected antibodies to SFV in 12% (8/69) of primatologists exposed to NWP, but failed to detect SFV DNA in those persons [29]. Hence, additional studies to further investigate the zoonotic potential of SFV from NWP are needed.

In the present report, we enrolled primate workers in a voluntary longitudinal, prospective study and collected their occupational primate exposure information. Blood and buccal swab specimens were collected and tested for evidence of infection with NWP SFV using previously validated, sensitive and specific serologic and PCR assays. We document persistent antibody to NWP SFV in these workers suggestive of chronic, persistent infection but also demonstrate seroreversion in others. We found no evidence of NWP SFV DNA sequences in any seroreactive workers.

## Material and methods

### Study population, sample collection and classification of NHP exposure risks

Workers occupationally exposed to NWP from Centro de Primatologia do Rio de Janeiro (CPRJ) and Fundação Jardim Zoológico da Cidade do Rio de Janeiro (RIOZOO) consented to participate in a study to investigate the occupational risk of simian retrovirus infection. Workers at CPRJ and RIOZOO were recruited to participate in the study following an information sharing presentation about SFV in NWPS and the exposure risks. All participants signed a consent form with information about this study and potential benefits of participation. The human subjects' research protocols were reviewed and approved by the Universidade Federal do Rio de Janeiro Review Board (54654616.0.0000.5257) and a project determination was approved for retroviral testing of these anonymized samples at CDC. Subjects were asked to answer a questionnaire describing type and length of exposure to NHPs (NWPs and OWPs) using a questionnaire adopted from a previous study of exposure to Old World monkeys and apes [13]. Specimens were collected at three different time points at each institution with multiple collections from most participants.

Risk for NHP exposure was classified into four levels based on type of contact. Level 1 included activities with indirect contact with simians, such as feeding and cage cleaning. Level 2 included activities with direct NHP contact but with no reported accidents, including capture and containment, medicine administration, blood collection, teeth extraction and cleaning, surgery and necropsy. Level 3 exposure included direct contact resulting in a reported accident, such as bites, scratches and injuries with sharp objects (scalpels, needles, etc.) containing simian body fluids. All workers without any reported NHP contact were classified as Level 0. Each individual was classified according to the highest contact risk level they reported.

### Sample preparation and confirmation of genomic DNA integrity

Ten milliliters of EDTA-treated whole blood was collected from each participant and refrigerated at 4°C for up to 8 hrs prior to processing. Plasma was separated from cells by centrifugation, aliquoted and stored at -80°C. Peripheral blood mononuclear cells (PBMCs) were isolated from whole blood with Ficoll-Paque^™^ Plus (GE Healthcare BioSciences, Pittsburgh, PA). Buccal swabs were collected using a cotton swab that was then placed immediately into a sterile tube containing 0.8% saline and stored at -20°C. Genomic DNA (gDNA) was extracted from PBMCs and oral swabs using the PureLink^®^ Genomic DNA kit (ThermoFisher Scientific, Grand Island, NY) following the manufacturer’s protocol and stored at -20°C. The integrity of the gDNA for PCR analysis was checked by PCR amplification of β-actin sequences using primers BAF1 (5’-GTG CTG TCC CTG TAC GCC TCT-3’) and BAR1 (5’-GGC CGT GGT GGT GAA GCT GTA-3’) as previously described [30]. All DNA samples testing positive for β-actin sequences were further considered suitable for SFV PCR detection.

### SFV serology

To insure detection of a broad range of genetically diverse NWP SFV, plasma samples were first screened for antibodies to NWP SFV using a Western blot (WB) assay containing antigens from both a marmoset (*Callithrix jacchus*, SFVcja) and spider monkey (*Ateles* species, SFVasp) as previously described [31, 32]. Briefly, plasma samples were diluted 1:50 and reacted separately to 150 μg of infected and uninfected cell lysates overnight at 4°C after protein separation through 4–12% polyacrylamide gels and transfer to Nytran membranes. Seroreactivity was detected using peroxidase-conjugated protein A/G (Pierce, Rockford, IL) and chemiluminescence (Amersham, Uppsala, Sweden). Seroreactivity to both Gag p68 and p72 precursor proteins with an absence of similar reactivity to antigen from uninfected Cf2Th cells was interpreted as seropositive. Samples with seroreactivity to a single Gag protein were considered seroindeterminate. Specimens without reactivity to either Gag protein were considered seronegative.

All participants with plasma samples reactive for NWP SFV were also tested by WB analysis for cross-reactivity to OWP SFV antigens derived from humans infected with SFVcsp (*Chlorocebus* species, African green monkey, previously referred to as SFVagm) and SFVptr (*Pan troglodytes*, chimpanzee, previously referred to as SFVcpz) and to SFV isolated from the prosimian *Galago crassicaudatus panganiensis* (SFVgal also known as SFVgpa) as described [33, 34].

### PCR analysis

All gDNA samples were first screened by PCR testing for detection of NWP SFV integrase sequences (192-bp) using a semi-nested approach that utilizes generic polymerase gene (*pol*) primers and conditions previously reported [35]. We refer to this test as the shorter *pol* PCR assay. Two additional NWP SFV subgenomic regions were targeted by nested PCR (398-bp LTR and *gag* sequences consisting of 225-bp in LTR and 173-bp in *gag*, and a 520-bp *pol* fragment). The latter is referred to as the longer *pol* PCR assay. Primers and PCR conditions for these additional fragments have been previously described [35]. PBMC DNA from persons with plasma seroreactivity to OWP SFV antigens were also tested using OWP SFV PCR analysis using nested PCR primers DNHF1/DNHR2 and DNHF3/DNHR4 to generate a 465-bp *pol* fragment as previously described (13).

We also applied a newly developed real-time quantitative PCR (qPCR) assay to simultaneously detect and quantify NWP SFV integrated in host gDNA of both PBMC and buccal swab specimens as described in detail elsewhere (Muniz et al., accepted PLoS ONE). Primers and probes were designed using an alignment of available *pol* sequences from NWP SFV, including representatives from all three NWP families [35]. Briefly, two forward and one reverse primers were used (QSIP4Nmod (for) 5’-TGC ATT CCG ATC AAG GAT CAG C-3’, QSIP4Nmod2 (for) 5’-YTT TGC YRC TTG GGC MAM RGA VA-3’, and QSIR1Nmod2 (rev) 5’- TTC CTT TCC ACY WTY CCA CTA CT-3’) with the probe DIAPR2 5’-FAM- TGG GGI TGG TAA GGA G”T”A CTG WAT TCC A-SpC6-3’, where “T” is labeled with the black hole (BHQ1) quencher and SpC6 is a six carbon spacer arm on the 3’ terminus to block polymerase activity by preventing the probe from priming. Following a 10 min incubation at 95°C to activate the Taq polymerase, a three step PCR was performed at 95°C for 15 sec, 50°C for 15 sec, and 62°C for 15 sec for 55 cycles using a BioRad CFX96 instrument.

Sensitivity of the qPCR assay was evaluated using replicative serial dilutions of seven different NWP SFV plasmids, including SFVcja, SFVasp, SFVcme (*Cacajao melanocephalus*, uakari monkey), SFVagu (*Alouatta guariba*, howler monkey), SFVssp (*Saimiri* species, squirrel monkey, also known as SFVsqu), SFVsxa (*Sapajas xanthosternos*) and SFVpsp (*Pithecia* species, saki monkey). The SFVcja and SFVssp plasmids were kindly provided by Joseph Sodroski (30). The SFVasp plasmids were generated by cloning of a *pol* PCR fragment amplified from infected tissue culture DNA from PBMCs of a spider monkey (*Ateles* species). The SFVcme, SFVagu, SFVsxa, and SFVpsp plasmids were generated by cloning of a *pol* PCR sequence amplified from PBMC DNA, all using the nested primers SNF3 and SNR3 as previously described (20). PCR fragments were cloned using the TA cloning kits per the manufacturer’s instructions (ThermoFisher Scientific, Grand Island, NY). Specificity of the qPCR assay was done by testing of PBMC gDNA from 35 human blood donors from the U.S. previously testing negative for OWP and NWP SFV (Muniz et al., accepted PLoS ONE) and HIV and HTLV. Additional specificity analysis of the SFV qPCR assay was done by testing of 30 NWPs from 12 different species (*Aotus nigriceps*, *Ateles paniscus*, *Brachyteles arachnoides*, *Callicebus personatus*, *Callithrix jacchus*, *Leontopithecus chrysomelas*, *Leontopithecus chrysopygus*, *Sapajus robustus*, *Sapajus apella*, *Cacajao melanocephalus*, *Pithecia monachus* and *Saimiri sciureus*) previously shown to be SFV WB and PCR negative as described (Muniz et al., accepted PLoS ONE).

To normalize the amount of diploid cells per reaction, a new generic qPCR assay was developed for the housekeeping gene ribonuclease P/MRP 30 kDa subunit (*RPP30*). Primers (RPP30FM 5’-GCA CAT TTG GAC CCT GCG AGC G-3’ and RPP30RM 5’-GTG AGC GGC TGT CTC CAC AAG-3’) and probe (RPP30PM 5’-HEX- TTC TGA CCT GAA GGC “T”CT GCG CGG-SpC6-3’) were designed using the human *RPP30* sequence (GenBank # U77665). The *RPP30* qPCR assay was performed using an initial 95°C incubation for 10 min followed by 55 cycles of 95°C for 15 sec and 62°C for 30 secs. The sensitivity of the *RPP30* qPCR assay was determined by using replicative serial dilutions of a positive control standard generated by PCR amplification of *RPP30* sequences using PBMC DNA from a pedigreed HIV, HTLV, and SFV-negative blood donor and the primers RPP30FM and RPP30RM and cloned as described above.

### Statistical analyses

The Mann—Whitney *U* test was used to evaluate an association between SFV NWP WB seropositivity and length of exposure to NWP. Workers classified with level 3 NHP exposure were divided into two groups, those with negative and positive SFV NWP WB results, which included two persons with indeterminate WB results. For length of exposure to NWP SFV, we considered the most recent time point at which the individual was seronegative in the NWM SFV WB assay. Statistical tests were considered significant at the level of *p* ≤ 0.05.

## Results

### Study population

Fifty-six workers occupationally exposed to NHPs at CPRJ (n = 18) and RIOZOO (n = 38) participated in our study (Table 1). Two workers at CPRJ and 12 at RIOZOO decided to not join the study. At CPRJ, blood samples were collected in 2011 from 15 participants, in 2013 from 18 persons and in 2014 from 11 workers. At RIOZOO specimens were collected in 2011 from 26 participants, in 2012 from 33 persons and in 2014 from 20 workers. Buccal swabs were collected in 2012 for 13 participants from CPRJ and 11 workers from RIOZOO and in 2014 from 11 persons at CPRJ and 20 workers from RIOZOO. Overall, we obtained serial blood specimens from 56 subjects (one sample from seven persons, two samples from 30 and three samples from 19 subjects) and serial buccal swabs from 40 subjects (one sample from 23 and two samples from 17 subjects). Nine volunteers performing laboratory research in Brazil consented to join the study, reported no contact with NHPs, and their blood samples were used as negative assay controls.

**Table 1:** Animal worker characteristics.

**Age**	
**Mean age in years (Standard deviation)**	41.1 (14.2)
**Median age in years**	41.5
**Gender** (**n = 56**)	
**Men**	86%
**Women**	14%
**Occupation** (**n = 56**)	
**Animal handler (percentage)**	19 (34%)
**Biologist**	3 (5%)
**Caretaker**	2 (4%)
**General helper**	10 (17%)
**Nurse**	2 (4%)
**Technician**	2 (4%)
**Trainee**	2 (4%)
**Veterinary**	7 (12%)
**Zoo technician**	1 (2%)
**No information (not provided)**	8 (14%)
**Exposure Profile** (**n = 56**)	
**Not exposed**	2
**Exposed only to NWP**	21
**Exposed only to OWP**	0
**Exposed to OWP and NWP**	26
**No information (not provided)**	7
**Level of NWP exposure**^a^ (**n = 56**)	
**Level 0**	2 (3.6%)
**Level 1**	8 (14.3%)
**Level 2**	6 (10.7%)
**Level 3**	33 (58.9%)
**No information (not provided)**	7 (12.5%)
**Individuals with accident reported** (**n = 56**)	33 (58.9%)
**Type of NWP level 3 exposure risk reported** (**accidents**)	
**Bite**	23 (37%)
**Scratch**	18 (29%)
**Body fluid contact**	18 (29%)
**Sharp injuries**	3 (5%)

^a^Levels of NHP exposure: level 0 included all workers without any reported NHP contact; level 1 included activities with indirect contact with simians; level 2 included activities with direct NHP contact but with no reported accidents and level 3 included direct contact resulting in a reported accident.

The mean age of the workers was 41 years old, with a high proportion of males (86%). Of nine different occupations reported, animal handler was the most common (34%) followed by general helper (17%). Eight individuals did not provide occupation information and seven did not provide information about NHP contact. At CPRJ, all 18 workers reported exposure to only NWPs, including *Alouatta guariba* (brown howler monkey), *Ateles species* (spider monkey), *Brachyteles arachnoides* (Southern muriqui), *Cacajao melanocephalus (Araca uakari)*, *Callithrix species* (marmoset), *Leontopithecus chrysomelas* (golden-headed lion tamarin), *L*. *chrysopygus* (golden-rumped lion tamarin), *L*. *rosalia* (golden lion tamarin), *Sapajus apella* (Margarita Island capuchin), *S*. *robustus* (crested capuchin), *Saguinus midas* (golden-handed tamarin) and *Saimiri sciureus* (common squirrel monkey). At RIOZOO, three workers reported exposure to only NWPs and 26 persons reported exposure to both NWPs and OWPs, including *Alouatta belzebul* (red-handed howler monkey), *A*. *guariba* (brown howler monkey), *A*. *seniculus* (Juruá red howler monkey), *Aotus species* (night monkey), *Ateles chamek* (black-faced black spider monkey), *Callicebus species* (titi monkey), *Callimico species* (Goeldii’s monkey), *Callithrix species* (marmoset), *Chiropotes species* (bearded saki monkey), *Lagothrix species* (woolly monkey), *Pithecia species* (saki monkey), *Saguinus fuscicalis* (saddleback tamarin), *Saimiri sciureus* (common squirrel monkey), *Sapajus apella* (Margarita Island capuchin), *S*. *flavius* (blonde capuchin), *S*. *robustus* (crested capuchin) and *S*. *xanthosternos* (yellow-breasted capuchin), *Chlorocebus sabaeus* (African green monkey), *Mandrillus sphinx* (mandrill), *Papio* species (baboon), *Pan troglodytes* (chimpanzee), *Pongo abelii* (Sumatran orangutan) and *Pongo species* (orangutan). Two persons at the RIOZOO reported no NHP exposures.

Interestingly, 59% (33/56) of the workers reported some type of accident involving NHPs with bites being most common (37%), followed by scratches and contact with body fluids (29%) and to a lesser extent sharp injuries (5%) (Table 1). Moreover, of the 33 workers who reported accidents, 33% (11/33) were animal handlers, 21% (7/33) were general helpers, 18% (6/33) were veterinarians, 9% (3/33) were biologists and 6% (2/33) were technicians. Caretakers, nurses, trainees and zoo technicians were 3% each (1/33 of each one).

### Detection of SFV seropositivity in workers occupationally exposed to NHP

**Fig 1:**
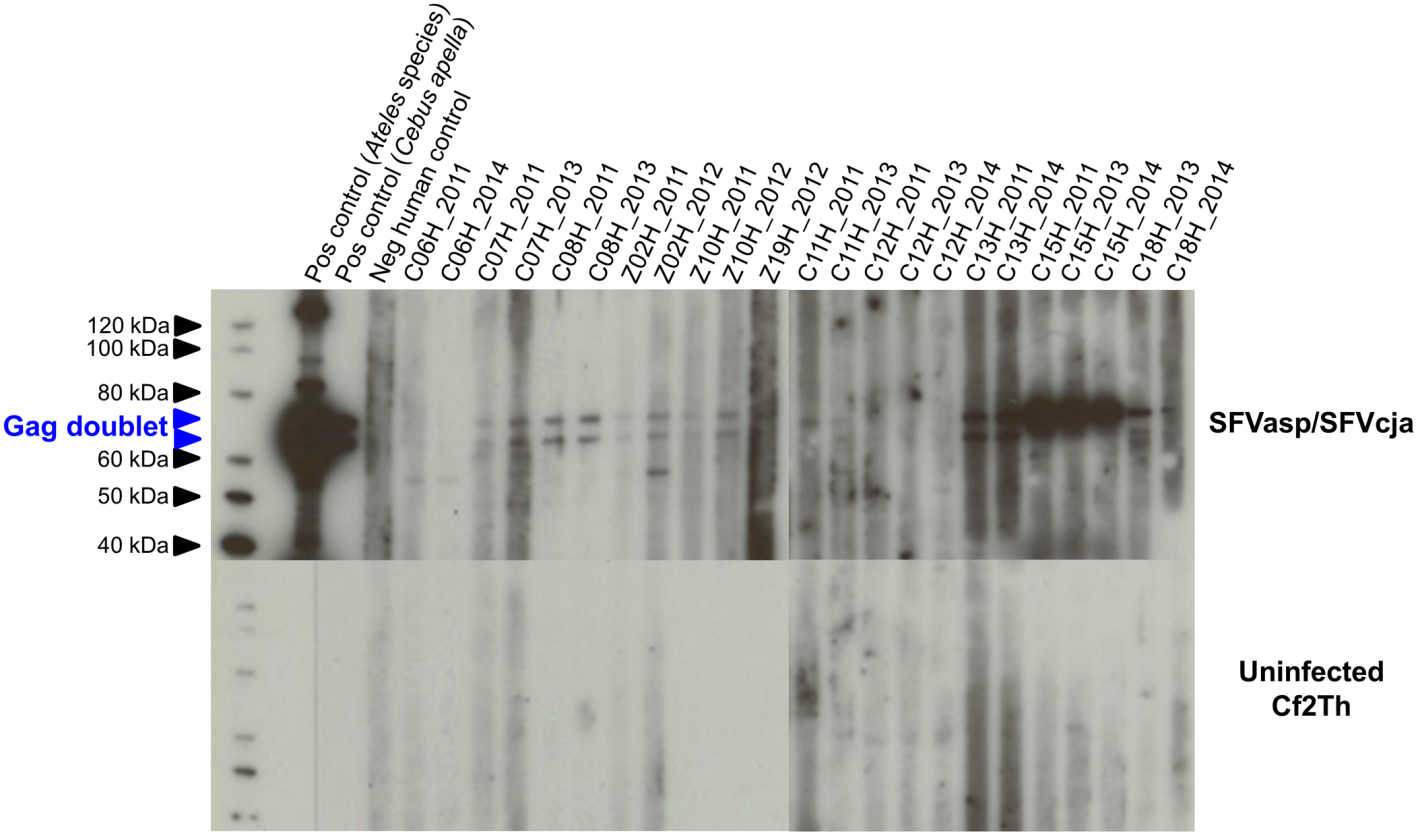
Detection of simian foamy virus (SFV) antibodies in workers exposed to New World primates. SFV antigens from a spider monkey (*Ateles* species, SFVasp) and a common marmoset (*Callithrix jacchus*, SFVcja) were prepared by expansion in canine thymocyte cells (Cf2Th) and were combined and reacted with test plasma and sera in the upper panel and simultaneously to uninfected Cf2Th antigens in the lower panel to check the specificity of the seroreactivity to the SFV antigens. Worker codes beginning with “C” and “Z” are from the Centro de Primatologia do Rio de Janeiro (CPRJ) and Fundação Jardim Zoológico da Cidade do Rio de Janeiro (RIOZOO), respectively. All human samples were reactive to both Gag proteins except for participants C15H and C18H whose samples were reactive for only the 72 kD Gag protein and which were classified as seroindeterminate. Two seropositive serum controls from an SFV-infected spider monkey (*Ateles* species) and a capuchin (*Cebus apella*) and a pedigreed seronegative human plasma sample (negative control) are included in each assay run. Molecular markers in kDa are provided on the left and the location of the 68/72 kDa Gag doublet proteins are shown with blue arrows.

Plasma samples were screened for antibodies to NWP SFV using a WB test that combines viral antigens from a spider monkey (SFVasp) and common marmoset (SFVcja). This WB assay has been shown to be highly sensitive and specific for detecting SFV in all three Platyrrhini families (33, 35). Results from a representative WB assay are shown in Fig 1. Plasma from 10/56 (18%) workers were found WB-positive for at least one collection time point (2011, 2012/2013 or 2014) (Table 2) showing reactivity to the diagnostic Gag doublet proteins p68 and p72). Five seropositive samples were from persons working at CPRJ, corresponding to 27.8% (5/18) of the workers, while WB seropositivity for workers at the RIOZOO was 13% (5/38). Occupations of the NWP SFV WB-positive workers included four general helpers (4/7, 40%), 3/7 (43%) veterinarians, and 3/18 (16%) animal handlers. For 12 NWP SFV seroreactive workers, seven (58.4%) reported accidents with only members of the *Cebidae* family, three (25%) with only the *Atelidae* family and two (16.6%) reported accidents with both NWP families (Table 2). Two samples (C15H and C18H) from CPRJ were classified as seroindeterminate showing strong seroreactivity to only a single Gag band (p72). All nine plasma samples from negative control individuals with no direct or indirect contact with NHPs were negative for NWP SFV antibodies.

**Table 2:** Case histories and simian exposures of New World primate (NWP) simian foamy virus (SFV)-seroreactive workers^a^.

*Worker*	*NWP WB*^b^ *status*	*OWP*^c^ *WB status*	*Prosimian WB status*	*Occupation*	*Duration of NHP exposure (years)*	*Risk level*^d^ *(0–3)*	*Accident Reported*	*Species involved in accident*
*C06H*	+	-	-	General helper	1.25	3	Bite	*Callithrix species*
*C07H*	+	-	-	General helper	1	3	Scratches, severe bite	*Leontopithecus rosalia*, *Saimiri sciureus*
*C08H*	+	-	-	General helper	1.16	3	Bite	*Leontopithecus chrysomelas*, *Brachyteles arachnoides*
*C11H*	+	-	-	General helper	1	3	Bite	*Brachyteles species*
*C13H*	+	-	-	Animal handler	8	3	Scratches	*Cebus apella*
*C15H*	Ind^e^	-	-	General helper	1	3	Scratches, bite	*Brachyteles arachnoides*
*C18H*	Ind	-	-	Caretaker	3	2	Feces contact	*Brachyteles arachnoides*
*Z02H*	+	-	-	Veterinarian	3	3	Bite, body fluid contact	*Callithrix jacchus*, *Cebus apella*
*Z10H*	+	-	-	Animal handler	20	3	Body fluid contact	*Cebus apella*, *Callithrix species*, *Papio species*, *Pan troglodytes*
*Z17H*	+	-	-	Veterinarian	29	3	Body fluid contact	*Cebus apella*, *Brachyteles species*, *Papio species*, *Pan troglodytes*
*Z19H*	+	-	-	Veterinarian	14	3	Bite, scratches, blood contact	*Cebus apella*, *Callithrix species*, *Papio species*, *Pan troglodytes*
*Z37H*	+	-	-	Animal handler	11	3	Scratches, body fluid contact	*Cebus apella*

^a^Worker codes beginning with “C” and “Z” are from the Centro de Primatologia do Rio de Janeiro (CPRJ) and Fundação Jardim Zoológico da Cidade do Rio de Janeiro (RIOZOO), respectively.

^b^WB, Western blot; WB status indicates positive/indeterminate (Ind) results at any time point (2011, 2012–2013, 2014). See Table 3 for details.

^c^OWP, Old World primate

^d^Risk level; 0, no reported simian contact; 1, indirect simian contact (feeding and cage cleaning); 2, direct contact but without reported injuries; 3, direct contact with reported injuries (bite, scratch, needle stick, etc.)

^e^Ind, indeterminate, seroreactivity to a single Gag protein

All 10 NWP SFV-positive and the two indeterminate plasma samples were subjected to additional WB analyses with OWP and prosimian SFV antigens to check for possible cross-reactivity. All 12 specimens tested negative in both the OWP and prosimian WB tests, including three workers (Z10H, Z17H and Z19H) having reported exposure to both NWP and OWP, but who were only reactive in the NWP WB tests (Tables 2 and 3).

**Table 3:** Longitudinal Western blot (WB) testing of workers for antibodies to simian foamy virus (SFV)^a^.

	*2011*	*2012/2013*	*2014*
*Worker*^b^	NWP^c^, OWP, PRO	NWP, OWP, PRO	NWP, OWP, PRO
*C06H*	w+^d^, -, -	-, -, ND^e^	-, -, -
*C07H*	+, -, ND	+, -, -	NA^f^, NA, NA
*C08H*	+, -, -	w+,—, -	NA, NA, NA
*C11H*	w+, -, -	w+, -, -	NA, NA, NA
*C13H*	+, -, -	+, -, -	+, -, -
*C15H*	Ind^g^, -, -	Ind, -, -	Ind, -, -
*C18H*	NA, NA, NA	Ind, -, -	Ind, -, -
*Z02H*	+, -, ND	+, -, -	-, -, -
*Z10H*	+, -, -	+, -, -	NA, NA, NA
*Z17H*	w+, -, -	w+, -, -	NA, NA, NA
*Z19H*	+, -, -	-, -, ND	NA, NA, NA
*Z37H*	NA, NA, NA	w+, -, -	+, -, -

^a^Specimens were collected at three time points in 2011, in 2012 or 2013, and in 2014.

^b^Worker codes beginning with “C” and “Z” are from the Centro de Primatologia do Rio de Janeiro (CPRJ) and Fundação Jardim Zoológico da Cidade do Rio de Janeiro (RIOZOO), respectively.

^c^NWP, New World primate WB; OWP, Old World primate WB; PRO, prosimian WB results

^d^w+, weak positive

^e^ND, not done

^f^NA, sample not available

^g^Ind, indeterminate, seroreactivity to a single Gag protein

Forty-four samples were not reactive to NWP SFV of which 32 were tested for antibodies to OWP SFV using specimens collected from at least one time point. Two samples (C04H and Z01H) were weakly OWP SFV-positive and three (Z05H, Z13H and Z14H) were indeterminate with reactivity to only a single band; all samples were collected in 2011. Worker C04H did not report contact with OWP but reported a bite accident by a *Leontopithecus chrysomelas* (golden lion tamarin). PCR testing of triplicate PBMC gDNA specimens from these five persons was negative for OWP SFV sequences, a finding that may represent cross-reactivity to NWP SFV or nonspecific seroreactivity.

### Contact risk levels and time of exposure to NHP

Workers were categorized into four contact risk levels based on severity of exposure to NHPs, with level 3 being the most severe and included bites, scratches and injuries with sharp objects (scalpels, needles, etc.) containing simian body fluids. All workers without any reported NHP contact were classified as Level 0. Each individual was classified according to the highest contact risk level they reported. All ten persons with NWP SFV WB-positive results were classified with level 3 contact risk (Table 2). Six of the 10 level 3 contact risk individuals reported being bitten by NWPs. Two persons (C15H and C18H) with indeterminate NWP SFV WB results were classified as level 3 and 2, respectively, and reported contact exclusively with NWPs (Table 2). Among 44 persons with NWP SFV WB-negative results, 22 (50%) were classified as level 3, five (11.3%) as level 2, eight (18.2%) as level 1 and two (4.5%) as level 0, while contact risk information was not provided for seven workers (16%) (Table 1). Two workers were WB-positive for OWP SFV; person C04H only reported contact risk level 3 exposure to NWP, while person Z01H reported contact risk level 3 exposure to OWP. Both workers were negative for OWP SFV sequences by PCR testing.

Thirty-three workers classified with level 3 exposures were further divided into two groups according to WB-positivity for NWP SFV and the length of historical contact with primates and were then compared by statistical analysis. Interestingly, the WB-negative persons (n = 21) had a median contact duration of 12.3 years, whereas the WB-positive workers (n = 12) had a median duration of NWP contact of 3 years. This difference was statistically significant (*p* = 0.033; Mann—Whitney *U* test). Of the 33 workers with level 3 exposures, six reported NWP contact exposure for less than 3 years. Five of these six persons (83.3%) were NWP SFV WB-positive and one (16.7%) was NWP SFV WB-negative. Of 12 workers with NHP contact exposure between 3–10 years, three (25%) were NWP SFV WB-positive and 9 (75%) were NWP SFV WB-negative. Fifteen persons reported contact exposure over 10 years, of which four (26.7%) were NWP SFV WB-positive and 11 (73.3%) were WB-negative. Thus, high-risk occupational exposures are common in this population and there appears to be an inverse correlation of NWP SFV seropositivity and duration of exposure to NWPs.

### Anti-SFV antibody clearance

Three of 10 (30%) workers who were initially NWP SFV WB-positive (C06H, Z02H and Z19H) did not retain seroreactivity at all three time points tested (Table 2, Fig 1). Samples from persons C06H and Z02H showed strong reactivity to both Gag precursor proteins in the WB assay at the first two collection time points (2011 and 2012/2013 (not shown)) but were WB-negative in 2014. The same pattern was observed for samples from person Z19H that showed reactivity only at the first time point, but not at the subsequent time point in 2012/2013. Samples from workers C15H and C18H remained seroindeterminate for NWP SFV reactivity at all three time points (Table 2, Fig 1).

### Nested PCR and qPCR for NWP SFV DNA in blood and buccal swabs

gDNA was extracted from PBMC samples from the 56 workers at the three different time points (a total of 124 PBMC gDNA samples) and was analyzed by nested PCR targeting three NWP SFV regions (shorter *pol* (192-bp), longer *pol* (520-bp) and LTR-*gag* primers) in order to confirm SFV infection molecularly and to allow identification of the virus by sequence analysis. All samples were tested in triplicate for each genomic region using 500 ng of gDNA. All 124 PBMC gDNA specimens were negative using the three different PCR tests. Given that our PCR assays are highly sensitive, that the assay controls performed as expected, and the quality of the extracted DNA was verified by PCR amplification of the β-actin gene, these results show that the 10 NWP SFV seropositive workers have undetectable levels of NWP SFV DNA in PBMCs.

Buccal swabs were also collected from persons who participated in the last two sample collections in 2012/2013 and 2014. Due to the low concentration of gDNA obtained from the buccal swabs, samples from some workers were not suitable for SFV PCR testing following the DNA quality PCR screening. For the 2012/2013 time point, the shorter *pol* PCR test was performed for 25 samples, whereas the longer *pol* region PCR was done for 17 samples, including all 10 NWP SFV WB-positive samples. Thirty-one buccal samples collected in 2014 were tested using the shorter and longer *pol* PCR assays, including all 10 specimens from the NWP SFV WB-positive persons. All buccal swab samples with sufficient gDNA amounts tested negative for *pol* sequences by nested PCR, corroborating the results obtained in the blood for those participants.

We validated the novel qPCR assay by determining the sensitivity of the assay to detect a broad range of highly genetically diverse NWP SFV from seven different NWP genera across all three NWP superfamilies: SFVcja (*Callithrix jacchus*), SFVasp (*Ateles* species), SFVcme (*Cacajao melanocehpalus*), SFVagu (*Alouatta guariba*), SFVssp (*Saimiri* species), SFVsxa (*Sapajas xanthosternos*), and SFVppi (*Pithecia pithecia*). The qPCR assay could reliably detect > 90% of replicates containing at least 20 copies of each SFV strain per μg DNA with all strains being detected at 100% of the 20 copy replicates except SFVcme which was detected 90% of the time at that concentration. All SFV control strains could be detected at five copies in 60–100% of the replicates, from 50–70% at three copies, and from 10–60% at a single copy. Assay specificity was verified by the absence of signal in PBMC DNA from 35 persons known to be negative for SFV, HIV, and HTLV using serology testing and by obtaining negative results using PBMC DNA from 30 SFV-seronegative diverse NWPs. All available PBMC gDNAs and buccal swab specimens from 2012/2013 and 2014 tested negative using the new qPCR assays that also targets *pol* sequences, indicating that if SFV is present in seropositive individuals the viral load is less than 20 copies/μg DNA. Insufficient amounts of gDNA from the 2011 PBMC specimens were available for qPCR testing.

## Discussion

Compared to OWP SFV, the potential of NWP SFV zoonotic transmission to humans is largely unknown. Recent studies have shown that SFV can be highly prevalent in both captive and wild NWPs, increasing potential infection risks for persons in contact with NWPs. In addition, the potential numbers and types of exposures to NWP SFVs are similar to those for OWP SFVs. For example, because of their relatively small size NWPs are cheaper to maintain and have simpler husbandry (high reproductive efficiency and can reach sexual maturity in about one year) and thus are frequently used in biomedical research, are common collections at zoological gardens, and are often kept as pets. An estimated 15,500–31,000 NWPs are used in research worldwide [27] and among 129 American Zoological Association (AZA) member institutions responding to a 2009 questionnaire, about 2,200 NWPs consisting of at least ten genera were at the zoos. NWPs are also frequently hunted. An estimated 2.2–5.4 million NWPs are hunted each year in the Brazilian Amazon alone [28]. Estimating numbers of monkeys kept as pets is difficult because these are illegal activities, are common among Amazonian river communities and are not typically reported. Thus, similar to OWP SFV, abundant opportunities exist for exposure and possible human infection with NWP SFV.

Until now only a single study has been published investigating the zoonotic potential of NWP SFV. In 2014 Stenbak *et al*. reported finding a WB seropositivity of 11.6% among 69 primatologists that reported exposure to NWP, and four of the eight SFV-positive subjects reported accidents involving NWP [29]. However, all eight seroreactive persons tested negative for NWP SFV sequences by PCR testing of blood specimens, which the authors explained could either represent latent infections occurring in other body compartments or viral replication controlled by host immune responses. In the present study, we found a higher serological prevalence (18%) to NWP SFV in primate workers using WB testing, considering results from at least one of three time points collected for each person. The greater prevalence found in our study may be attributed to a possible higher exposure of our population to NWP, with a high number (59%) of workers reporting accidents involving NWP body fluids, including bites and scratches. However, the total number of persons reporting accidents involving NWP in the former study were not reported for direct comparison [29]. We also used a prospective study design with longitudinal sampling of the workers over a three-year period which may have permitted detection of additional positives not found by a cross-sectional study design. This study design also allowed us to detect persistent SFV antibody in some individuals over a period of two to three years, suggestive of possible ongoing viral replication.

All ten NWP SFV-positive individuals in our study reported accidents with NWP. Interestingly, six of these workers were bitten by NWP, reaffirming other evidence that biting is very efficient for SFV transmission [17]. All ten NWP SFV-positive samples were not seroreactive to OWP and prosimian SFV, showing further the specificity of our WB assay. Two individuals (C15H and C18H) were seroindeterminate in the NWP SFV WB test at all time points. These two workers reported scratches/bites and contact with feces, respectively, for the same NWP species, *Brachyteles arachnoides* (wooly spider monkey). While reactivity to a single Gag protein band may reflect a specific antibody response to SFV from this primate species, it is atypical of other SFV infections where reactivity to both Gag proteins is seen. For example, another worker (C11H) also reported bite exposures to *Brachyteles arachnoides*, but showed reactivity to the characteristic double Gag bands. Therefore, additional factors such as nonspecific seroreactivity to the NWP SFV antigens might explain the seroindeterminate WB pattern observed in these two persons. Additional serologic testing such as measurement of antibody titers and/or neutralizing antibodies may help to further characterize these infections but adequate plasma volumes were not available for this testing.

PCR assays targeting four viral regions, three by nested PCR and one using qPCR, were used to detect NWP SFV sequences in gDNA prepared from both PBMC and buccal swabs of the seroreactive workers [35, 36]. However, we did not detect NWP SFV sequences in any sample. Nonetheless, our findings are consistent with those of Stenbak *et al*. [29] and our combined findings suggest that human infection with NWP SFV may persist with extremely low SFV viral DNA loads in both the blood and the oral mucosa. These results contrast with those obtained from humans infected with OWP SFV, in which proviral sequences can be consistently detected in PBMCs in most cases using nested PCR and qPCR and also in oral specimens [13, 14, 20, 37]. For example, we have previously detected SFV DNA in PBMCs from all nine persons with those specimens and in saliva from three of seven SFV-positive humans occupationally exposed to NHP by nested PCR and isolated SFV from the oral cavity from a person infected with a chimpanzee SFV [13, 20]. More recently Rua *et al*. showed that SFV DNA, but not RNA, could be detected by nested PCR and qPCR in PBMCs in all 14 hunters infected with ape SFV but in only 8/14 (57.1%) saliva samples from these persons [21]. Proviral loads in these persons were very low (< 20 copies/10^5^ PBMCs) which is close to the assay limit of detection of three copies/150,000 PBMCs used in that study and may explain why virus was not detected in all compartments of all persons by qPCR [21]. Detectable viral loads in the oral mucosa in that study were associated with a long duration of infection (median > 16 yrs.) but were present in less than five SFV DNA copies/10^5^ cells [21]. In contrast, persons in our study with seroreactive NWP SFV results reported primate exposures for an estimated median duration of less than three years, which could help explain the negative PCR results in the oral fluids. The sensitivity of our qPCR assay was determined to reliably detect 20 copies/ug DNA (about 150,000 cell equivalents) though the assay could also detect from one to five copies/ug DNA for some NWP SFV strains, like that in other studies, but less frequently. It’s possible that the NWP SFV DNA levels in infected humans is lower than what can be reliably detected by qPCR or nested PCR.

Little is also known about viral loads in infected NWPs. For example, Stenbak *et al*. found that proviral loads in blood specimens from two squirrel monkeys were as low (177 copies/150,000 PBMCs) as those in four OWPs (macaques) and a human infected with a macaque SFV [29]. Testing of NHP buccal swabs were not reported in that study for comparison with our results [29]. Thus, more studies are needed to investigate SFV expression and replication sites in NWP and in humans to better interpret the lack of proviral sequence detection in seropositive persons.

The undetectable NWP SFV proviral loads in exposed humans may also reflect a latent or well-controlled infection. The activity of human restriction factors that mediate innate immunity against retroviruses may play in a role in controlling this infection. TRIM5α proteins, for example, mediate a species-specific blockage of infection by particular retroviruses. Pacheco *et al*. showed that squirrel monkey SFV replication was inhibited by human TRIM5α, whereas marmoset and spider monkey SFV were not inhibited [38]. Other restriction factors, like tetherin and APOBEC3G, were shown to inhibit FV replication but their specific activity on NWP SFV is still undetermined [39, 40]. It is plausible that these intrinsic factors modulate NWP SFV infection in humans by stabilizing the virus and maintaining very low proviral loads. FV are also potent inducers of type I interferon (IFN) by plasmacytoid dendritic cells and by PBMC, and such activation of the innate immune responses may be involved in the control of SFV replication in humans [41]. Alternatively, the undetectable viral levels in blood and buccal specimens and longitudinal detection of SFV antibodies may be from sequestration and active replication of virus in lymph nodes or some other tissue compartment.

We also found serological evidence of antibody clearance after a first seropositive result for NWP SFV in three workers over a two- to three-year period. These results suggest possible immune control of SFV infection in these workers. To investigate seroreversion in these persons further we also compared the duration of exposure to NWP between the NWP SFV WB-positive and negative workers in our study and found a significant difference in length of exposure and seropositivity in these two groups. NWP SFV WB-positive individuals had an overall shorter duration of NWP exposure compared with seronegative workers, though some SFV-positive workers were consistently seroreactive and had decades of exposure. Although this is the first evidence of antibody clearance in SFV infection, other retroviruses, such as simian type D retrovirus (SRV), can present as latent infections with the presence of integrated virus detected by PCR and/or tissue culture but without antibody detection and with each SRV analyte waxing and waning overtime without consistent detection [42]. Antibody clearance could also reflect possible abortive infections. A complete understanding of these results will require further studies, including additional follow-up of SFV-seropositive persons for longer periods of time.

In summary, using a prospective study design we examined a group of workers occupationally exposed to Neotropical primates in Brazil to better understand the zoonotic transmission potential of SFV infecting these monkeys. We used validated serological assays, and nested PCR and qPCR assays which are highly sensitive and specific for detecting infection with NWP SFV. We identified a high prevalence of seropositive workers against NWP SFV, documenting human susceptibility to these viruses and show that none had detectable levels of SFV DNA reflecting latent or well-controlled infections. We also document for the first time antibody reversion in several NWP SFV WB-positive workers, suggesting possible latent infection or clearance of infection. Our findings contribute to the current understanding of human infection with SFV from NWP.

## Supporting information

S1 File Study questionnaire. This file contains the study questionnaire in Portuguese and translated into English.
(DOCX)
