# Supplementary material for: Zoonotic infection of Brazilian primate workers with New World simian foamy virus
Source: PLoS One. 2017 Sep 20;12(9):e0184502. doi: 10.1371/journal.pone.0184502 (PMC5606925; doi:10.1371/journal.pone.0184502)
Supplement: S1 File — This file contains the study questionnaire in Portuguese and translated into English. (DOCX) [file pone.0184502.s001.docx]

Questionário (Portuguese)

Data da coleta: __/__/____

Nome do participante:___________________________________________

Data de nascimento: __/__/___

Telefone de contato: ________________

1. Qual das alternativas abaixo corresponde a sua ocupação no trabalho?
2. Veterinário
3. Pesquisador
4. Tratador
5. Técnico em pesquisa
6. Outro: __________________
7. Qual o seu sexo?

a) Feminino b) Masculino

1. Por quanto tempo você trabalhou ou trabalha com macacos? ______________
2. Com quais macacos tropicais você teve contato? (circule)

4.1) Macaco prego

4.2) Macaco prego do peito amarelo

4.3) Macaco barrigudo ou macaco peludo

4.4) Sagui

4.5) Macaco-aranha

4.6) Macaco da noite

4.7) Mico-leão da cara dourada

4.8) Carinha Preta (Mico-leão da cara preta)

4.9) Acari

4.10) Macaco de cheiro

4.11) Caiarara

4.12) Sauá

4.13) Cuxiú, macaco preto

4.14) Uacari

4.15) Muriqui

4.16) Guariba, bugio

Outros:____________________________________________________________________________________________________________________________________

5) Circule as atividades que você já exerceu ou exerce no seu trabalho com primatas tropicais. Por quanto tempo você desempenhou ou desempenha tal atividade (preencha o tempo nas lacunas)?

1. Alimentação _________________________
2. Limpeza de gaiolas ____________________
3. Captura e retenção ___________________
4. Administração de medicamentos ____________________
5. Coleta de sangue ______________________
6. Limpeza ou extração de dentes ___________________________
7. Cirurgia _________________________________
8. Autopsia _________________________________
9. Outros _________________________________________________________

6) Enquanto você trabalhava com primatas tropicais, você alguma vez: (circule)

1. Foi mordido
2. Foi arranhado
3. Foi ferido por agulha ou outros instrumento de trabalho
4. Teve contato com fluidos corporais ou tecido do animal

Caso você tenha circulado alguma(s) das opções acima, escreva qual espécie o acidente aconteceu:

__________________________________________________________________________________________________________________________________________

7) Com quais macacos africanos ou asiáticos você teve contato? (circule)

7.1) Colobus

7.2) Macaco Resus

7.3) Chimpanzé

7.4) Orangotango

7.5) Babuíno

7.6) Gorila

7.7) Mandril

7.8) Mangabei

7.9) Bonobo

7.10) Outros: ________________________

8) Circule as atividades que você já exerceu ou exerce no seu trabalho com primatas africanos ou asiáticos. Por quanto tempo você desempenhou ou desempenha tal atividade (preencha o tempo nas lacunas)?

1. Alimentação _________________________
2. Limpeza de gaiolas____________________
3. Captura e retenção ___________________
4. Administração de medicamentos ____________________
5. Coleta de sangue ______________________
6. Limpeza ou extração de dentes ___________________________
7. Cirurgia _________________________________
8. Autopsia _________________________________
9. Outros _________________________________________________________

9) Enquanto você trabalhava com primatas africanos (Velho Mundo), você alguma vez: (circule)

1. Foi mordido
2. Foi arranhado
3. Foi ferido por agulha ou outros instrumento de trabalho
4. Teve contato com fluidos corporais ou tecido do animal

Caso você tenha circulado alguma(s) das opções acima, escreva qual espécie o acidente aconteceu:

__________________________________________________________________________________________________________________________________________

Questionnaire (English)

Date of collection : __/__/____

Name of participant:___________________________________________

Date of birth: __/__/___

Phone number: ________________

1. Which of the following is your occupation at work?
2. Veterinarian
3. Researcher
4. Animal handler
5. Research Technician
6. Other: __________________
7. What is your gender?

a) Female b) Male

1. How long have you worked with monkeys? ______________
2. What tropical monkeys did you have contact with? (Circle)

4.1) Capuchin monkey

4.2) Yellow breasted capuchin monkey

4.3) Common Woolly Monkey

4.4) Marmoset

4.5) Spider monkey

4.6) Night (Owl) monkey

4.7) Golden lion tamarin

4.8) Black lion tamarin

4.9) Saki monkey

4.10) Squirrel monkey

4.11) White fronted capuchin

4.12) Titi monkey

4.13) Bearded saki monkey

4.14) Uakari

4.15) Southern muriqui

4.16) Brown howler

Others:____________________________________________________________________________________________________________________________________

5) Circle the activities you have already done or practice in your work with tropical primates. How long have you performed or perform such an activity (fill the gaps)?

1. Feeding _________________________
2. Cleaning cages ____________________
3. Capture and detention ___________________
4. Medication administration ____________________
5. Blood collection ______________________
6. Cleaning or extraction of teeth ___________________________
7. Surgery _________________________________
8. Autopsy _________________________________
9. Others _________________________________________________________

6) While you worked with tropical primates, have you ever been: (circle)

1. Bitten
2. Scratched
3. Injured by needle or other work tool
4. Has had contact with body fluids or animal tissue, If you have circled any of the above, write down what kind of accident happened:

__________________________________________________________________________________________________________________________________________

7) What African or Asian monkeys did you have contact with? (Circle)

7.1) Colobus

7.2) Rhesus monkey

7.3) Chimpanzee

7.4) Orangutan

7.5) Baboon

7.6) Gorilla

7.7) Mandrill

7.8) Mangabey

7.9) Bonobo

7.10) Others: ________________________

8) Circle the activities you have already done or practice in your work with African or Asian primates. How long have you performed or perform such an activity (fill in the gaps)?

1. Feeding _________________________
2. Cleaning cages ____________________
3. Capture and detention ___________________
4. Medication administration ____________________
5. Blood collection ______________________
6. Cleaning or extraction of teeth ___________________________
7. Surgery _________________________________
8. Autopsy _________________________________
9. Others _________________________________________________________

9) While you worked with African (Old World) primates, have you ever been: (circle)

1. Bitten
2. Scratched
3. Injured by needle or other work tool
4. Has had contact with body fluids or animal tissue, If you have circled any of the above, write down what kind of accident happened:

__________________________________________________________________________________________________________________________________________
